# Iodoform Intoxication

**Published:** 1884-03

**Authors:** 


### Iodoform Intoxication.

We have been hearing a good deal of late about poisoning from the absorption of iodoform applied locally, and there already exists sufficient evidence to make us careful in its use. Mr. P. J. Hayes reports a case in the *Dublin Jour. Med. Science,* August, 1883, and makes some remarks on the subject. In the mild cases there is anorexia, headache, loss of memory; moroseness or even delirium; pulse accelerated and temperature elevated. In grave cases, hallucinations or furious delirium, urine scanty, temperature perhaps 104°, and in fatal cases, death in a state of coma. He concludes by saying:

"I think few surgeons will be found to oppose the recommendation of Martin and Veliaminoff, that iodoform should, in all cases where its use is indicated, be applied in small quantity; that especial caution should attend its use when much adipose tissue is exposed, and when the wound is extensive; that its employment is more or less contra-indicated by advanced age, tendency to fatty degenerations, diseased conditions of heart or lungs, and when there exists a susceptible condition of the nerve centres.'

